# The Reformed Public House

**Published:** 1924-02

**Authors:** Fred Stewart

**Affiliations:** 36, Victoria Street, Westminster, S.W. 1


Sir,—Why should anyone object to the extension of the Carlisle scheme advocated by Mr. Dering? As to the cost, which appears to alarm Mr. Simons, the scheme could be extended gradually, which would involve Treasury advances in payment, or as a national scheme. This would not involve the country in any financial outlay, because the transfer to the State would be effected by the exchange of Government bonds for brewery securities. A profitable business would be secured by the State and the influence of a great monopoly would cease, to the great advantage of the nation. The weakness of Mr. Simons's argument that it is doubtful if the success attained by Carlisle could be maintained under a national scheme is obvious. Why, if it is a success there, should it not be equally successful elsewhere?

Again, if there are already many examples of what public houses should be, why do not the brewery owners bring all their houses to the standard they have themselves created? There is evidently no need to wait for facilities, because Mr. Simons admits that it has been done. The fact is, of course, that the liquor trade has betrayed its trust. For the sake of profits it has turned the business into a huge money-making concern by concentrating on the sale of beer, regardless of its responsibility and undertaking to cater for the public need. The truth is that the existence of the Carlisle scheme has frightened the brewery companies, and here and there tentative efforts have been made to improve the houses. There is nothing to stop them from completing the work—they have had a free hand for centuries, and, as Mr. Dering has pointed out, the People's Refreshment House Association and similar bodies can provide suitable places. Why not the Trade? The facilities that the Trade seeks are in the direction of increasing the sale of beer, and this the authorities are not disposed to grant. But this should be no bar to the brewery companies providing decent refreshment houses. S. JOHN LONGMAN.

SIR,—Your correspondent, Mr. A. P. Grubb, advocates local option, combined with a form of disinterested management. Does he realise that by the adoption of his suggestion the voters of a particular area will be empowered, in the event of a disinterested management option being carried, to vote away millions of public money on a purely local investment? This proposal is contrary to every principle of sound finance, and is, in the present state of the country, an impractical one.

FRED STEWART.

36, Victoria Street,
Westminster, S.W. 1.

